# A victorivirus infecting *Colletotrichum falcatum*: genomic characterization and evolutionary analysis

**DOI:** 10.1007/s00705-026-06611-7

**Published:** 2026-03-24

**Authors:** Maressa O. Henrique, Diego Z. Gallan, Augusto Schrank, Renata O. Dias, Robert H. A. Coutts, Ioly Kotta-Loizou, Marcio C. Silva-Filho

**Affiliations:** 1 Departamento de Genética, Escola Superior de Agricultura Luiz de Queiroz, Universidade de São Paulo, Av. Pádua Dias, 11, Piracicaba, 13418-900 SP Brasil; 2 Departamento de Biologia Molecular e Biotecnologia, Universidade Federal do Rio Grande do Sul, Porto Alegre, RS Brasil; 3 Departamento de Genética, Instituto de Ciências Biológicas, Universidade Federal de Goiás, Goiânia, GO Brasil; 4 Department of Life Sciences, Faculty of Natural Sciences, Imperial College London, London, SW7 2AZ UK; 5 Department of Clinical, Pharmaceutical & Biological Science, School of Health, Medicine and Life Sciences, University of Hertfordshire, Hatfield, AL10 2AB UK

## Abstract

*Colletotrichum falcatum* is a major fungal pathogen responsible for severe disease in sugarcane. Here, we report the complete genomic characterization of a novel mycovirus infecting *C. falcatum*, designated Colletotrichum falcatum victorivirus 1 (CfVV1). The CfVV1 genome is 4,906 base pairs (bp) long and comprises two open reading frames encoding a capsid protein (CP) and an RNA-dependent RNA polymerase (RdRP), overlapping at an AUGA motif. The predicted proteins share ca. 70% and 68% identity with those of Colletotrichum cliviicola victorivirus 1 and Colletotrichum nativitas totivirus 1, respectively. Phylogenetic analysis based on concatenated RdRP and CP amino acid sequences places CfVV1 within the genus *Victorivirus* of the recently established family *Pseudototiviridae*. Transmission electron microscopy confirmed the presence of isometric virus-like particles ca. 40 nm in diameter, consistent with the typical morphology of victoriviruses. To our knowledge, this is the first report of a complete victorivirus genome infecting *C. falcatum*.

Mycoviruses are viruses that infect fungi across nearly all major taxonomic groups, including economically important plant-pathogenic species, and coevolve with their host cells, like a cellular organelle, showing great biological diversity [1, 2, 3]. Despite their diversity, most described mycovirus families in the International Committee on Taxonomy of Viruses (ICTV; ictv.global/taxonomy) are associated with double-stranded RNA (dsRNA) genomes. Currently, these dsRNA mycoviruses are classified into 17 families encompassing 35 genera and 303 species (https://ictv.global/virus-properties;accessed on 16/07/2025).

Recently, the ICTV reorganized the former family *Totiviridae* into two distinct families, *Pseudototiviridae* and *Orthototiviridae*. The new family *Pseudototiviridae* comprises four genera: three previously established genera *Leishmaniavirus*,*Trichomonasvirus*, and *Victorivirus* formerly included in *Totiviridae* together with the newly created genus *Eimeriavirus*. Conversely, the previously established genus *Totivirus* has been reassigned to the new family *Orthototiviridae* [4]. Members of the genus *Victorivirus*, now placed within the family *Pseudototiviridae*, are characterized by their strict fungal host range, the presence of isometric virions ca. 40 nm in diameter, and a non-segmented RNA genome ranging in size from 4.6 to 6.3 kbp. Typically, this genome encodes only a capsid protein (CP) and an RNA-dependent RNA polymerase (RdRP), with expression occurring through a stop/restart translation mechanism [5, 6].

The fungus *Colletotrichum falcatum* Went (teleomorph = *Glomerella tucumanensis* (Speg.) Arx & Müll.) was first isolated from sugarcane in 1892 and is recognized as the causal agent of red rot disease [7, 8]. Members of the genus *Colletotrichum* (phylum Ascomycota) rank among the top ten plant pathogens of greatest scientific and economic importance and are responsible for red rot symptoms in a close association with a major insect pest, *Diatraea saccharalis* (Lepidoptera: *Pyralidae*) in sugarcane, leading to severe agricultural losses [9–13]. In this study, we describe a novel mycovirus, Colletotrichum falcatum victorivirus 1 (CfVV1), identified in *C. falcatum* strain 01/18, isolated from sugarcane in Brazil. Genome organization and phylogenetic analyses suggest that CfVV1 represents a new member of the genus *Victorivirus*, family *Pseudototiviridae*.

*C. falcatum* strain 01/18 was isolated from stalk tissue of sugarcane cultivar CTC13 (*Saccharum* ssp.) at the Centro de Cana-IAC (Sugarcane Centre of the Agronomic Institute) in Ribeirão Preto, São Paulo, Brazil. Molecular identification was carried out by sequencing the internal transcribed spacer (ITS) region and the glyceraldehyde-3-phosphate dehydrogenase (GAPDH) gene (data not shown). The fungus was maintained on potato dextrose agar (PDA) plates at 25 ± 3 °C in the dark and stored long-term in 25% (v/v) glycerol at -80 °C. For dsRNA extraction and purification, *C. falcatum* mycelial plugs were inoculated into potato dextrose broth (PDB) and incubated at 25 °C for 72 h with shaking at 120 rpm. Filtered mycelia were homogenized in liquid nitrogen and ground into a fine powder. Cell lysis was performed in sodium chloride-Tris-EDTA buffer (STE), 1% (w/v) sodium dodecyl sulfate (SDS), and 0.1% (v/v) β-mercaptoethanol, followed by nucleic acid extraction with an equal volume of phenol: chloroform: isoamyl alcohol (25:24:1). The dsRNA was purified by chromatography on a cellulose fiber (Sigma-Aldrich^®^) column using STE buffer with 16% (v/v) ethanol, as described previously [14, 15]. To remove DNA and single-stranded RNA (ssRNA) contaminants, the dsRNA was treated with RNase-free DNase I (Thermo Fisher Scientific) and S1 Nuclease (Promega), respectively. The nuclease-treated dsRNA was then electrophoresed on a 0.8% (w/v) agarose gel containing 0.1% (v/v) SYBR™ Safe DNA Gel Stain (Invitrogen) and visualized under ultraviolet (UV) light using an L-PIX transilluminator (Loccus).

To provide direct evidence for the presence of viral particles, CfVV1 particles were purified from 20 g of powdered mycelium. Following an adapted protocol [16], the homogenate was clarified with chloroform and butanol, precipitated with 8% PEG 6000, and concentrated by ultracentrifugation. Purified virions were negatively stained and examined under a Zeiss EM 900 transmission electron microscope.

The purified dsRNA was sequenced using the Illumina NovaSeq platform (Macrogen, Republic of Korea). The viral genome was assembled using SPAdes v3.13.1 [17, 18]. Final contigs were queried using the Basic Local Alignment Search Tool (BLAST) x against the non-redundant (nr) protein database at National Center for Biotechnology Information (NCBI). Sequencing gaps were filled by OneStep RT-PCR (Qiagen) amplification using primers based on the identified cDNA sequences. Amplicons corresponding to the gap sequence were cloned into the pGEM-T Easy plasmid (Promega) and transformed into *Escherichia coli* strain DH5α cells for Sanger sequencing with M13 primers. Manual assembly of overlapping fragments using BioEdit Sequencer v7.7.1 reconstructed the full-length cDNA sequence.

Potential open reading frames (ORFs) were predicted by the NCBI ORFfinder and the relative molecular mass (*Mr*) of the predicted proteins was calculated using Bioinformatics.org molecular weight calculator. RdRP and CP amino acid (aa) sequences encoded by 48 established species of family *Pseudototiviridae* (retrieved from ICTV) as well as homologous victoriviruses sequences from other *Colletotrichum* spp. from the NCBI database were aligned by Multiple Alignment using Fast Fourier Transformation (MAFFT) version 7.526 with L-INS-i algorithm [19]. Ambiguously aligned sites were removed by trimAl version 1.5 with “strict” mode [20]. The two aligned genes were concatenated using FASconCAT-G [21] and a maximum likelihood phylogenetic tree was constructed using IQ-TREE version 3.0.1 with the best-fit model recommended by ModelFinderPlus algorithm (Q.PFAM + F+G4 for CP and Q.PFAM + F+I+R6 for RdRP) and 1000 ultrafast bootstrap replicates [22–24]. The phylogenetic tree was visualized using the Interactive Tree of Life (iTOL) web server v6 [25].

*C. falcatum* strain 01/18 contains the undivided dsRNA genome (Fig. 1A) of CfVV1, which belongs to the genus *Victorivirus* in the family *Pseudototiviridae*. The complete genome sequence of CfVV1 has been deposited in the GenBank database under the accession number PX911346. The CfVV1 genome has a GC content of 60% and is 4,906 bp in length flanked by 5’ and 3’ untranslated regions (UTRs) that are 278 and 72 bp in length, respectively. Two large ORFs were identified using the NCBI ORFfinder (Fig. 1B). ORF1, which encodes the CP, is 2,097 bp (698 aa long) in length, with a predicted *Mr* 72.95 kDa, whereas ORF2 encodes the RdRP and is 2,463 bp (820 aa long) in length, with a predicted *Mr* 89.35 kDa.

To corroborate the genomic data, transmission electron microscopy was performed on partially purified preparations from *C. falcatum* strain 01/18. Spherical, isometric virus-like particles, ca.40 nm in diameter, were readily observed (Fig. 1C), which is consistent with the typical morphology of members of the genus Victorivirus. This provides direct physical evidence for the presence of CfVV1 virions.

The two ORFs overlap and share a tetranucleotide motif (AUGA), in which the stop codon of ORF1 (UGA) overlaps the initiation codon of ORF2 (AUG), spanning nucleotide (nt) positions 2,372 to 2,375. Analysis of the terminal 42 bp of ORF1 (CP) reveals a highly stable H-type pseudoknot (ΔG = -16.76 kcal/mol at 25 °C) predicted by the KnotInFrame algorithm and visualization performed using FORNA (ViennaRNA Web Services) [26, 27]. AUGA is a key motif for the translation of the second ORF, acting in conjunction with a pseudoknot signal located within a 32 nt region immediately upstream of the AUGA motif [28, 29], a feature that is characteristic of victoriviruses [30–33]. The ORFs were compared against the NCBI non-redundant (nr) protein database using BLASTp. The CP showed 73.28% aa identity to the CP of Colletotrichum cliviicola victorivirus 1 (CcVV1; Accession PP944850) and 70.45% aa identity to the CP of Colletotrichum nativitas totivirus 1 (CnTV1; Accession MK279492). Similarly, the RdRP showed 70.00% aa identity to the RdRP of CcVV1 and 68.95% aa identity to the RdRP of CnTV1. Motif searches against the NCBI Conserved Domain Database (CDD) revealed that CfVV1 contains a conserved CP domain (Totivirus_coat, pfam05518) in ORF 1 and a conserved RdRP domain (RdRP_4, pfam02123) in ORF2.

The CfVV1 RdRP shares 70.00% aa identity with that of CcVV1, despite the two viruses being isolated from distinct host species. According to the current species demarcation criterion for the genus *Victorivirus*, which is based on RdRP aa sequence identity (< 70%), CfVV1 and CcVV1 fall near the threshold, supporting their classification as closely related yet potentially distinct viruses [4]. In addition, their genomic organization differs in their stop and initiation signals: CcVV1 uses the predicted UAAUG motif (where the UAA stop codon of ORF1 overlaps the AUG start codon of ORF2), whereas CfVV1 uses predicted AUGA motif (where the UGA stop codon of ORF1 overlaps the AUG start codon of ORF2).

The C-terminus of the CfVV1 CP contains an Ala/Gly/Pro-rich (A/G/P; 62.5%) region spanning from aa 667–698, which is also a characteristic feature of victoriviruses’ CPs [4, 6]. Furthermore, multiple alignment of the predicted RdRP aa sequences of CfVV1 and representative members of the family *Pseudototiviridae* revealed the presence of eight conserved motifs (I–VIII) (Fig. 1D). Recently, the order *Ghabrivirales*, which comprises mycoviruses with dsRNA genomes, was reorganized based on consensus aa sequences of three core RdRP motifs (A, B, and C), resulting in three new suborders: *Alpha*-, *Beta*- and *Gammatotivirineae* [4]. The family *Pseudototiviridae* was established within the suborder *Alphatotivirineae*, and its members are characterized by the presence of motifs A, B, and C, which is found in conserved motifs IV, V, and VI, respectively.

**Fig. 1:**
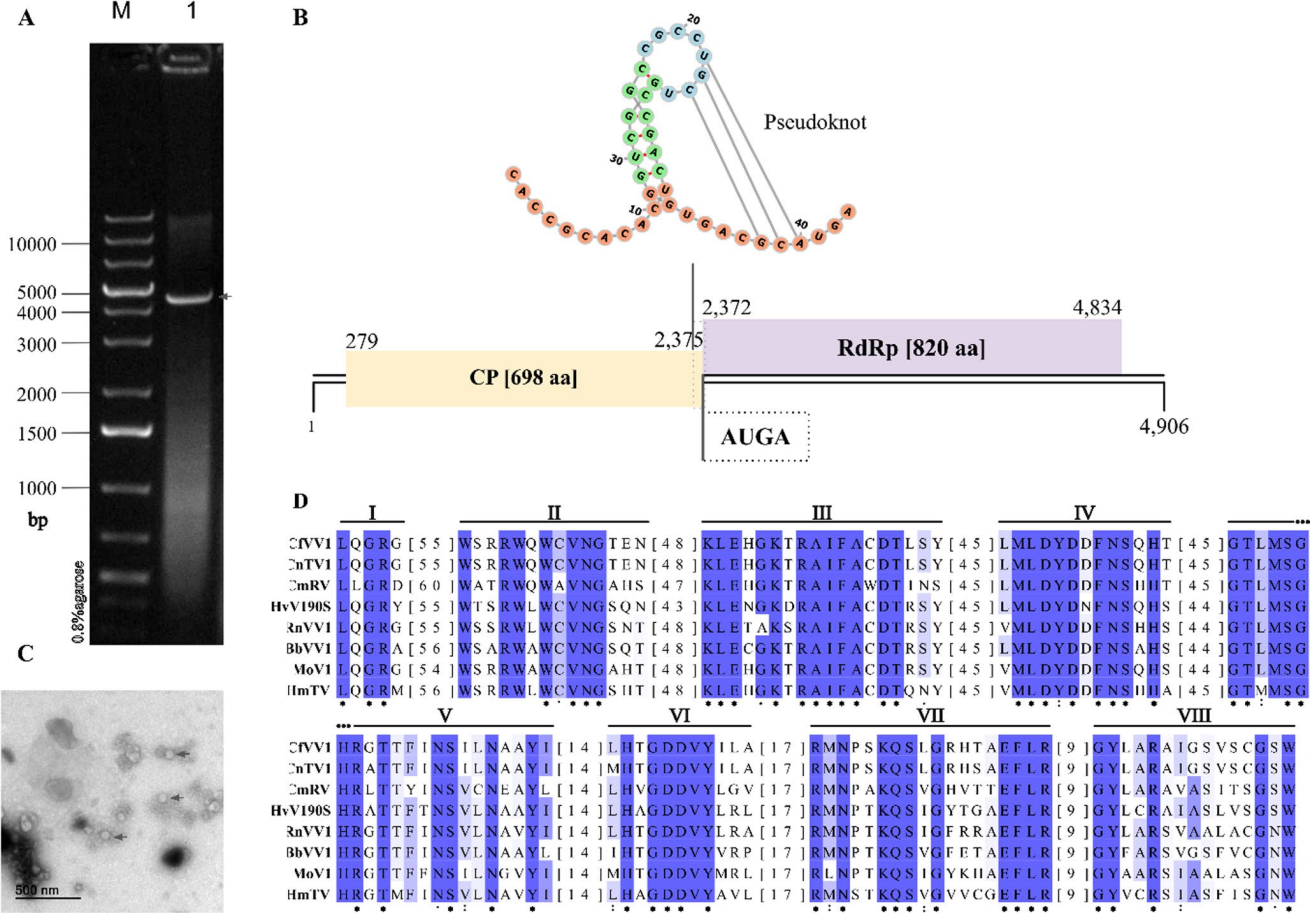
Genomic features of the Colletotrichum falcatum victorivirus 1 (CfVV1). (A) Agarose gel (0.8%) electrophoresis of viral dsRNA (lane 1) extracted from *C. falcatum* strain 01/18 after DNase I and S1 nuclease digestions and 1 kb Plus DNA molecular weight marker (Thermo Scientific; lane M). Arrow indicates the CfVV1 genomic dsRNA. (B) Predicted secondary structure of pseudoknot (bases 22–24 pairing with 38–40) of the ORF1/ORF2 junction region and schematic representation of the CfVV1 genome; the genome is 4906 bp in length and contains two ORFs which encode a CP and an RdRP, respectively. The black lines indicate the 5′- and 3′-UTR regions. The yellow and purple boxes indicate predicted ORFs and nt positions are indicated. (C) Transmission electron micrograph of purified CfVV1 particles. Arrows indicate the viral particles. Scale bar = 500 nm. (D) Alignment of conserved RdRP motifs aa sequences. Conserved motifs (I-VIII) typically found in RdRPs highlighted in purple. Along with CfVV1, RdRP sequences from Colletotrichum nativitas totivirus 1 (CnTV1; Accession MK279492), Coniothyrium minitans RNA virus (CmRV; Accession AF527633), Helminthosporium victoriae virus 190 S (HvV190S; Accession U41345), Rosellinia necatrix victorivirus 1 (RnVV1; Accession AB742454), Beauveria bassiana victorivirus 1 (BbVV1; Accession HE572591), Magnaporthe oryzae virus 1 (MoV1; Accession AB176964), and Helicobasidium mompa totivirus 1–17 (HmTV; Accession AB085814), were also included in the alignment. Identical aa residues are indicated by asterisks (*), conserved and semi-conserved residues are indicated by colons (:) and periods (.), respectively. Numbers in brackets indicate the sequence length between motifs

Phylogenetic analyses were performed to clarify the taxonomic placement of CfVV1. A phylogenetic tree based on RdRP and CP aa sequences was inferred using the maximum-likelihood method (Fig. 2). The results showed that CfVV1 clustered together with members of the genus *Victorivirus*, separate from the other genera of the family *Pseudototiviridae*. Based on its genomic characteristics and phylogenetic relationships, we propose CfVV1 as a new member of the genus *Victorivirus* in the new family *Pseudototiviridae*. To the best of our knowledge, CfVV1 is the first victorivirus reported to infect *C. falcatum*.

**Fig. 2:**
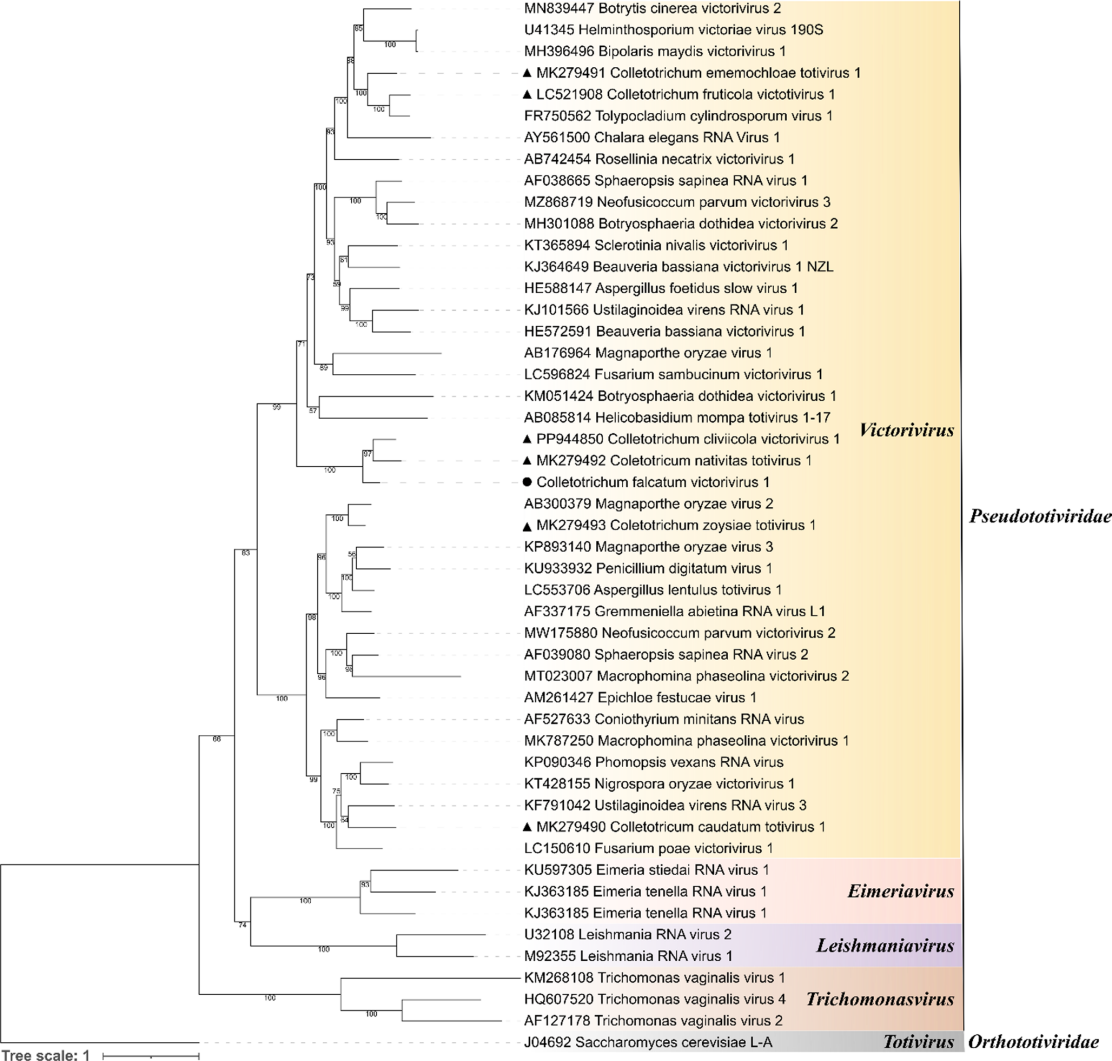
Phylogenetic analysis of RdRP and CP of Colletotrichum falcatum victorivirus 1 (CfVV1)**.** Phylogenetic tree inferred based on the alignment of RdRP and CP aa sequences using the maximum likelihood method. The black triangle (▲) indicates Colletotrichum falcatum victorivirus 1 from our study and black circles (●) indicate victorivirids sequences from other *Colletotrichum* spp. Tree branches with bootstrap probability values less than 50% based on 1000 replicates are hidden. A virus belonging to genus *Totivirus*, family *Orthototiviridae*, was used as the outgroup

## Data Availability

The genomic sequence determined in this study was submitted to NCBI under the accession number PX911346.

## Declarations

### **Supporting.**

National Institute of Science and Technology (INCT) in Ecology, Evolution, and Biodiversity Conservation, funded by CNPq (grant 465610/2014-5/ 409197/2024-6) and FAPEG (grant 201810267000023).
